# Confirmation of *BIK* and *SAMHD1* as Prostate Cancer Susceptibility Genes

**DOI:** 10.1002/pros.70037

**Published:** 2025-08-18

**Authors:** Christian P. Pavlovich, Jun Wei, Marta Gielzak, Zhuqing Shi, Huy Tran, Annabelle Ashworth, S. Lilly Zheng, Patrick C. Walsh, Jun Luo, Brian T. Helfand, William B. Isaacs, Jianfeng Xu

**Affiliations:** ^1^ James Buchanan Brady Urological Institute Johns Hopkins School of Medicine Baltimore Maryland USA; ^2^ Program for Genomic Translational Research Endeavor Health Evanston Illinois USA; ^3^ Division of Urology Endeavor Health Evanston Illinois USA; ^4^ University of Chicago Pritzker School of Medicine Chicago Illinois USA

**Keywords:** AOX1, BIK, FAM111A, germline, mutation, prostate cancer, SAMHD1

## Abstract

**Background:**

To independently assess data for recently reported genes—*BIK*, *SAMHD1*, *FAM111A*, and *AOX1*—in which rare variants have been associated with prostate cancer (PCa) risk and aggressiveness.

**Methods:**

The study included 4448 PCa cases from Johns Hopkins School of Medicine and 103,221 population‐based controls from the Genome Aggregation Database (gnomAD). Gene‐based and variant‐based association tests were performed within each major ancestry group using Fisher's exact test and Firth logistic regression. Bonferroni‐corrected significance thresholds were applied to account for multiple testing.

**Results and Conclusion:**

In the NFE population, suggestive statistical evidence of association with PCa risk was found for two of the four genes, *BIK* and *SAMHD1*. The evidence was primarily driven by missense variants: *BIK* S87G and *SAMHD1* Q465K and V112I. The effect sizes of these variants were stronger than, or comparable to, those of well‐established PCa risk variants such as *HOXB13* G84E and *CHEK2* T367fs. A weak association signal was observed between *AOX1* and PCa aggressiveness. Statistical power was limited for analyses in other ancestry groups, particularly for tests of PCa aggressiveness. Implication of *BIK* and *SAMHD1* as PCa susceptibility genes represents a major breakthrough since the discovery of *HOXB13* in 2012 and may have clinical utility for risk stratification and contribute to our understanding of the molecular etiology of PCa.

## 1 Introduction

Two recently published studies identified several genes—*BIK*, *SAMHD1*, *FAM111A*, *AOX1*, among others—in which rare variants are significantly and strongly associated with increased risk of prostate cancer (PCa) and its aggressiveness [1, 2]. Notably, both studies included large sample sizes, providing substantial statistical power to detect associations of rare variants. The first study by Ivarsdottir and colleagues analyzed 21,697 PCa cases and 320,527 controls from population‐based cohorts in Iceland, the UK, and Norway [1], while the second study by Mitchell and colleagues included 19,926 PCa cases and 187,705 controls from UK and Mexico population cohorts, as well as participants from a series of AstraZeneca clinical trials [2]. Both studies employed rigorous designs, including the use of a stringent significance threshold based on Bonferroni correction to control for multiple testing. Importantly, two of the genes (*BIK* and *SAMHD1*) were implicated in both studies and are involved in biological pathways distinct from those of many previously established PCa susceptibility genes that are primarily involved in DNA damage response [3, 4, 5, 6].

However, as with all genetic discovery studies and as cautioned by both papers, the associations of these genes with PCa risk and aggressiveness require confirmation in independent study populations. This is particularly important given the substantial overlap in study subjects between the two studies, with approximately half of the PCa cases and controls in both studies drawn from the same UK Biobank cohort [1, 2]. Furthermore, all significant associations in both studies are susceptible to population stratification bias, where observed associations may be driven by differences in genetic ancestry between cases and controls rather than true disease‐related variants [7]. Rare variants are particularly sensitive to the effects of population stratification because they typically are unique to a particular population's ancestry (founder variants) [8].

In this study, we evaluated associations for these newly reported PCa genes and variants in independent study populations. We performed association tests within each ancestry group to reduce potential population stratification.

## 2 Methods

The PCa patients were from the Brady Urological Institute and Comprehensive Cancer Center at the Johns Hopkins School of Medicine, Baltimore, MD (hereafter referred to as Hopkins). The majority of these PCa patients were enrolled from those who underwent radical prostatectomy for treatment of clinically localized PCa since 1987 [9]. Pathological evaluation and tumor grading were consistently performed by Hopkins pathologists. Radical prostatectomy cases with preoperative PSA ≥ 20, pathological Gleason score ≥8, pathologic pT3b, N1, metastatic disease, or who suffered death from PCa were classified as aggressive PCa, otherwise, as nonaggressive PCa. Only patients with available germline whole‐exome sequencing and SNP array data were included in the study. Genetic ancestry probabilities were estimated using the top 20 principal components (PCs) derived from 16,109 ancestry informative markers across the genome from SNP arrays. Individuals were classified as Non‐Finnish European (NFE), Ashkenazi Jewish (ASJ), or African/African American (AFR) if their estimated genetic ancestry probability was ≥75% for the corresponding group. The study cohort was approved by the Institutional Review Boards at Johns Hopkins School of Medicine.

Control subjects were drawn from the Genome Aggregation Database (gnomAD, version 4.1), a collection of public datasets that aggregates and harmonizes genomic and exome sequencing data from large‐scale projects [10]. As gnomAD may include individuals with cancer, including PCa, they were considered as population controls. To ensure independence from the two previously published studies that utilized the UK Biobank (UKB) cohort [1, 2], we excluded all UKB individuals from the gnomAD data set for the current analysis. Furthermore, to match genetic ancestry of cases, only controls from NFE (*N* = 93,944), ASJ (*N* = 5573) and AFR (*N* = 3704) were included.

Confirmatory association tests were performed for four genes and 10 rare variants reported in the two published studies [1, 2]. Given that the reported genes and variants were primarily identified in individuals of European ancestry—and considering that rare variants are often ancestry‐specific—the primary analysis was conducted in the NFE population, with secondary analyses performed in the ASJ and AFR populations. For gene‐based analysis, aggregated carrier rates of variants in a gene between two groups (case vs. controls or aggressive vs. nonaggressive cases) were compared. Following the approaches used in the prior studies [1, 2], four types of variants were analyzed: (1) synonymous variants (Synonymous), served as a negative control, (2) protein truncating variants (PTVs), including stop gain, stop loss, splicing, and frameshift variants, (3) rare missense changes with carrier rate <1% (Missense), and (4) all nonsynonymous variants, including PTVs, Missense, and inframe insertions/deletions (Non‐Synonymous). Variant‐based association tests were limited to rare variants with carrier rate <1% in the NFE population controls. Given the low frequency of these variants, the Fisher's exact test and the Firth logistic regression model were used. The Fisher's exact test was used for case‐control analyses due to the lack of individual data from gnomAD controls. The Firth logistic regression model was used for case‐case analyses where individual data were available for both aggressive and nonaggressive patients, and the analyses were performed adjusting for age and the top 10 PCs. To control for multiple testing, Bonferroni‐corrected significance thresholds were applied: *p* < 0.0125 for gene‐based tests (4 primary tests in NFE) and *p* < 0.005 for variant‐based tests (10 tests in NFE), ensuring a study‐wise error rate of <5%.

## 3 Results and Discussion

Key characteristics of 4448 PCa patients from Hopkins are presented in sTable 1, including 1317 and 3063 patients with aggressive and nonaggressive disease, respectively. Control subjects in the gnomAD (*N* = 103,221) by ancestry populations are also presented. Germline variants identified in the four genes (*BIK*, *SAMHD1*, *FAM111A*, and *AOX1*) from the Hopkins and gnomAD cohorts are listed in sTable 2.

In the primary confirmatory gene‐based analysis for PCa risk in the NFE population, which included 3193 cases and 93,944 controls, none of the four genes reached the predefined significance threshold (*p* < 0.0125) (Table 1). However, a trend toward increased PCa risk was observed for *SAMHD1* and *BIK* across several variant models, particularly the model including missense variants only [*SAMHD1*, odds ratio (OR) = 1.50, *p* = 0.03; *BIK*, OR = 1.56, *p* = 0.06]. In both genes, PTVs were extremely rare—2 carriers for *SAMHD1* and 1 carrier for *BIK*—and had lower rates in cases than controls. In contrast, most observed variants in these genes were missense changes, which showed approximately 1.5‐fold higher aggregated carrier rates in cases than in controls. Notably, two recurrent missense variants in *SAMHD1* were significantly and strongly associated with increased PCa risk at *p* < 0.05: Q465K (5 carriers) had an OR of 4.6, *p* = 0.007, and V112I (7 carriers) had an OR of 2.4, *p* = 0.03 (Table 2a). Similarly, one recurrent missense variant in *BIK* (S87G, 5 carriers) was also significantly and strongly associated with PCa risk, with an OR of 9.82, *p* = 0.0004 (Table 2b), confirming the finding reported by Ivarsdottir and colleagues [1]. In the secondary confirmatory gene‐based analysis among individuals of ASJ and AFR ancestry, no significant associations were found for any of the four genes. These findings are consistent with the original reports [1, 2], in which *BIK* and *SAMHD1* were the only genes associated with PCa susceptibility in both studies, where the majority of subjects were of European ancestry.

**TABLE 1:** Gene‐based association tests for prostate cancer risk.

		NFE				ASJ				AFR			
		Carriers, No. (%)				Carriers, No. (%)				Carriers, No. (%)			
Gene	Variants	Cases *N* = 3193	Controls *N* = 93,944	OR (95% CI)	p**	Cases *N* = 495)	Controls *N* = (5573)	OR (95% CI)	p**	Cases *N* = 760)	Controls *N* = (3704)	OR (95% CI)	p**
*AOX1*	Synonymous	49 (1.53)	1744 (1.86)	0.82 (0.61–1.10)	0.20	7 (1.41)	87 (1.56)	0.90 (0.35–1.96)	1.00	14 (1.84)	97 (2.62)	0.70(0.37–1.24)	0.25
	PTVs	14 (0.44)	457 (0.49)	0.9 (0.49–1.53)	0.80	0 (0.0)	1 (0.02)	0 (0–436.10)	1.00	1 (0.13)	14 (0.38)	0.35(0.01–2.29)	0.49
	Missense	75 (2.35)	2222 (2.37)	0.99 (0.78–1.25)	1.00	14 (2.83)	137 (2.46)	1.15	0.55	24 (3.16)	187 (5.05)	0.61 (0.38–0.95)	0.02
	All nonsynonymous*	89 (2.79)	2695 (2.87)	0.97 (0.77–1.20)	0.83	14 (2.83)	138 (2.48)	1.15 (0.61–2.02)	0.65	25 (3.29)	202 (5.45)	0.59 (0.37–0.90)	0.01
*FAM111A*	Synonymous	16 (0.50)	612 (0.65)	0.77 (0.44–1.26)	0.37	7 (1.41)	81 (1.45)	0.97 (0.38–2.11)	1.00	28 (3.68)	100 (2.7)	1.38 (0.97–2.13)	0.15
	PTVs	3 (0.09)	162 (0.17)	0.54 (0.11–1.62)	0.38	0 (0.0)	0 (0.0)	nan	1.00	0 (0.0)	0 (0.0)	nan	1.00
	Missense	40 (1.25)	1164 (1.24)	1.01 (0.72–1.39)	0.94	8 (1.62)	71 (1.27)	1.27 (0.53–2.67)	0.53	11 (1.45)	57 (1.54)	0.94 (0.44–1.82)	1.00
	All nonsynonymous*	43 (1.35)	1335 (1.42)	0.95 (0.68–1.29)	0.82	8 (1.62)	71 (1.27)	1.27 (0.53–2.67)	0.53	11 (1.45)	57 (1.54)	0.94 (0.44–1.82)	1.00
*SAMHD1*	Synonymous	13 (0.41)	335 (0.36)	1.14 (0.59–1.96)	0.65	0 (0.0)	1 (0.02)	0 (0–436.10)	1.00	10 (1.32)	52 (1.4)	0.94 (0.42–1.87)	1.00
	PTVs	2 (0.06)	64 (0.07)	0.92 (0.11–3.46)	1.00	0 (0.0)	4 (0.07)	0 (0–17.08)	1.00	0 (0.0)	6 (0.16)	0 (0–4.14)	0.60
	Missense	34 (1.06)	671 (0.71)	1.50 (1.03–2.12)	0.03	1 (0.2)	8 (0.14)	1.41 (0.03–10.54)	0.54	6 (0.79)	27 (0.73)	1.08 (0.36–2.69)	0.82
	All nonsynonymous*	36 (1.13)	746 (0.79)	1.42 (0.99–1.99)	0.04	1 (0.2)	12 (0.22)	0.94 (0.02–6.36)	1.00	6 (0.79)	33 (0.89)	0.89 (0.30–2.14)	1.00
*BIK*	Synonymous	6 (0.19)	199 (0.21)	0.89 (0.32–1.97)	1.00	6 (1.21)	77 (1.38)	0.88 (0.31–2.01)	1.00	1 (0.13)	2 (0.05)	2.44 (0.04–46.88)	0.43
	PTVs	1 (0.03)	36 (0.04)	0.82 (0.02–4.86)	1.00	0 (0.0)	2 (0.04)	0 (0–60.06)	1.00	0 (0.0)	1 (0.03)	0 (0‐189.51)	1.00
	Missense	19 (0.60)	358 (0.38)	1.56 (0.93–2.48)	0.06	0 (0.0)	16 (0.29)	0 (0–2.92)	0.64	6 (0.79)	17 (0.46)	1.73 (0.56–4.61)	0.26
	All nonsynonymous*	31 (0.97)	755 (0.8)	1.21 (0.81–1.74)	0.31	3 (0.61)	63 (1.13)	0.53 (0.11–1.64)	0.37	11 (1.45)	47 (1.27)	1.14 (0.53–2.25)	0.72

*All nonsynonymous variants include PTVs, Missense, and inframe insertions/deletions.

*Fisher's exact test.

**TABLE 2:** Variant‐based association tests for prostate cancer risk in the NFE population.

		Carriers, No. (%)			
Genes	Variants	Cases (*N* = 3193)	Controls (*N* = 93,944)	OR	*p*
**a**					
* SAMHD1*	c.G334A:p.V112I	7 (0.22)	86 (0.09)	2.4	0.0338
* SAMHD1*	c.C1393A:p.Q465K	5 (0.16)	32 (0.03)	4.6	0.0069
**b**					
* HOXB13*	c.G251A:p.G84E	35 (1.10)	379 (0.40)	2.74	4.81E‐07
* CHEK2*	c.1100delC:p.T367fs	29 (0.91)	435 (0.46)	1.97	0.001
* BIK*	c.A259G:p.S87G	5 (0.16)	15 (0.02)	9.82	0.0004
* BIK*	c.416_445del:p.139_149del	6 (0.19)	137 (0.15)	1.29	0.48
* WNT9B*	c.G454A:p.E152K	5 (0.16)	170 (0.18)	0.87	1
* SP2*	c.A612G:p.T204T	23 (0.72)	761 (0.81)	0.89	0.69
* CDK5RAP3*	17‐47971193‐G‐A (intron)	0 (0.00)	641 (0.68)		1
* CDK5RAP3*	c.A1367G:p.Q456R	23 (0.72)	768 (0.82)	0.88	0.62
* ABI3*	c.51G>T:p.E17D	0 (0.00)	8 (0.01)	0	1
* COL1A1*	c.2414C>T:p.P805L	0 (0.00)	5 (0.01)	0	1

*Fisher's exact test.

We also tested the associations of these four genes with PCa aggressiveness using a case‐case analysis in the NFE group of the Hopkins cohort, which included 1057 and 2095 patients with aggressive and nonaggressive disease, respectively (sTable 3). No significant association was found for any of these genes, likely due in part to limited statistical power of small sample sizes. However, a nonsignificant association with PCa aggressiveness was found for *AOX1* where carrier rates were higher in aggressive cases than in nonaggressive cases across several variant models, especially for PTVs (OR = 1.50, *p* = 0.46). This finding is consistent with previous studies where *AOX1* was the only gene reported to be associated with aggressive PCa, OR = 2.60, *p* = 1.36E‐06 [2]. No association test for PCa aggressiveness was performed in minority ancestry groups due to limited sample sizes.

Variant‐based confirmatory association tests for PCa risk were performed in the NFE population for 10 rare variants previously implicated in either of the two published studies [1, 2]. Three variants met the prespecified significance threshold (*p* < 0.005), including *HOXB13* G84E, *CHEK2* T367fs, and *BIK* S87G (Table 2b). The first two are well‐established PCa risk variants [11, 12, 13], while *BIK* S87G is located in a gene implicated in PCa risk in both prior studies [1, 2]. Notably, all five carriers of the *BIK* S87G variant in the Hopkins cohort shared a minimum 833 kb haplotype containing the variant (chr17:42,822,283–43,655,427) and were not genetically related (kinship coefficients < 0.01). This shared haplotype suggests that S87G is a founder variant, likely of Icelandic origin, given that its frequency is approximately tenfold higher in Icelanders compared to individuals from the UK and Norway [1]. No supporting evidence was observed for the remaining seven variants (all *p* > 0.05). One of these is another recurrent *BIK* variant (p.139_149del), a 30 bp in‐frame deletion involving a trinucleotide (TCG) repeat. Although its association with PCa was not statistically significant (OR = 1.29, *p* = 0.48) [2], the variant is of poor quality due to challenges in calling variants within this microsatellite repeat region [1]. The remaining six variants are located in genes that have not been previously associated with PCa in other studies, including those by Ivarsdottir et al. and Mitchell et al. [1, 2]

Overall, results from gene‐based and variant‐based association tests in the NFE population of our study yield several key findings: (1) suggestive evidence supporting two of the four newly identified PCa susceptibility genes (*BIK* and *SAMHD1*); (2) confirmation of a novel PCa risk variant (*BIK* S87G); (3) suggestive evidence for two rare recurrent missense variants in *SAMHD1* (Q465K and V112I); and (4) a weak association signal between *AOX1* and PCa aggressiveness.

The discovery and replication of *BIK* and *SAMHD1* are notable, given that few major PCa susceptibility genes have been identified and consistently replicated since the discovery of *HOXB13* in 2012 [11]. Genes with relatively strong effects on PCa risk (OR > 2) and moderately common risk alleles in the general population (>0.3%), such as *BRCA2*, *ATM*, *CHEK2*, and *HOXB13*, have likely already been captured through earlier case‐control studies [9]. Larger studies, such as those by Ivarsdottir et al. and Mitchell et al., are essential for detecting additional risk genes harboring rare variants, especially when stringent significance thresholds are required to correct for multiple testing [1, 2]. Another important factor contributing to the identification of *BIK* is the use of founder populations, in which certain disease‐associated variants are enriched but extremely rare in populations of more diverse ancestry backgrounds [1]. For example, the allele frequency of *BIK* S87G is 0.104% in the Icelandic population, eightfold higher than that in the UK Biobank and Norwegian cohorts (0.013%) [1]. The value of founder populations for genetic discovery is further illustrated by the recent identification of the ASJ founder variant *MMS22L* F772fs, which was associated with PCa risk [14].

The identification of these novel PCa susceptibility variants has potential clinical implications for PCa risk assessment. The ORs associated with the three implicated variants—9.82 for *BIK* S87G, 4.6 for *SAMHD1* Q465K, and 2.4 for *SAMHD1* V112I—are high, exceeding or comparable to those of established PCa risk variants such as *HOXB13* G84E (OR = 2.74) and *CHEK2* T367fs (OR = 1.98). However, the association data for these variants with PCa aggressiveness are limited at this stage due to the small sample size. The proportions of aggressive PCa were 0%, 60%, and 0% among the 5, 5, and 7 carriers of *BIK* S87G, *SAMHD1* Q465K, and *SAMHD1* V112I, respectively.

Importantly, the discovery of these two novel PCa susceptibility genes may improve our understanding of molecular mechanisms underlying PCa risk, extending beyond the well‐established DNA damage response pathways. For instance, *BIK* encodes a proapoptotic protein that can suppress tumor growth by promoting apoptosis in PCa cells [15]. mRNA levels of *BIK* in 49 human tissues from the Genotype‐Tissue Expression project (GTEx, v.v8) reveal that *BIK* is expressed highly in prostate tissue [1]. The missense variant S87G is in an annotated splice region and likely leads to loss of the BH3 domain of the BIK protein [1]. SAMHD1, a deoxynucleotide triphosphate (dNTP) hydrolase, plays key roles in DNA replication and cell cycle regulation and may also act as a negative regulator of innate immune responses and a facilitator of DNA end resection during DNA replication and repair [16]. Both Q465K and V112I variants are located within the catalytic core domain of the protein [17]. In vitro studies have demonstrated that four other missense variants within this domain (V133I, A338T, R366H, and D497Y) identified in colon cancer patients, significantly reduced or abolished dNTPase activity, leading to imbalanced dNTP pools [18]. In vivo, hemizygous *SAMHD1*⁺/^−^ embryos exhibited elevated and imbalanced dNTP pools compared to wild‐type controls [18].

It is important to note that the statistical power of our study to confirm associations for genes harboring rare risk variants is limited, particularly in minority ancestry populations. For example, the power to detect significant gene‐based associations (*p* < 0.0125) for genes with low aggregated carrier rates (e.g., 0.5%) and moderate effect sizes (OR of 2) was estimated at only 2.5%, 1.6%, and 1.7% for the NFE, ASJ, and AFR groups, respectively. These limitations emphasize the need for caution when interpreting null findings and highlight the inherent challenges in detecting rare variant associations. Another major limitation of this study is the lack of functional validation for the implicated variants, which is necessary to confirm their biological relevance and mechanistic impact on prostate cancer risk.

In conclusion, our study confirmed several rare PCa risk variants in two newly reported genes, which may have clinical utility for risk stratification and contribute to a deeper understanding of the molecular etiology of PCa.

## Conflicts of Interest

J. Xu serves as a scientific advisory board member for GoPath Diagnostics and GenomicMD.

## Supporting information


**sTable1:** Clinical phenotypes in the Hopkins cohort.
**sTable 2:** Identified variants in 4 genes.
**sTable 3:** Gene‐based association tests for prostate cancer aggressiveness.

## Data Availability

The data that support the findings of this study are available from the corresponding author upon reasonable request.
